# Cell-Based Manufacturing Technology Increases Antigenic Match of Influenza Vaccine and Results in Improved Effectiveness

**DOI:** 10.3390/vaccines11010052

**Published:** 2022-12-26

**Authors:** Steven Rockman, Karen Laurie, Chi Ong, Sankarasubramanian Rajaram, Ian McGovern, Vy Tran, John Youhanna

**Affiliations:** 1 CSL Seqirus Ltd., Parkville, VIC 3050, Australia; 2 Department of Immunology and Microbiology, The University of Melbourne, Parkville, VIC 3050, Australia; 3 CSL Seqirus Ltd., Maidenhead SL6, UK; 4 CSL Seqirus Ltd., Summit, NJ 07901, USA; 5 CSL Seqirus Ltd., Kirkland, QC H9H 4M7, Canada

**Keywords:** influenza vaccines, mammalian cell-based influenza vaccines, antigen match, egg-based influenza vaccines, egg adaptation, recombinant influenza vaccines, hemagglutinin, neuraminidase, effectiveness

## Abstract

To ensure that vaccination offers the best protection against an infectious disease, sequence identity between the vaccine and the circulating strain is paramount. During replication of nucleic acid, random mutations occur due to the level of polymerase fidelity. In traditional influenza vaccine manufacture, vaccine viruses are propagated in fertilized chicken eggs, which can result in egg-adaptive mutations in the antigen-encoding genes. Whilst this improves infection and replication in eggs, mutations may reduce the effectiveness of egg-based influenza vaccines against circulating human viruses. In contrast, egg-adaptive mutations are avoided when vaccine viruses are propagated in Madin-Darby canine kidney (MDCK) cell lines during manufacture of cell-based inactivated influenza vaccines. The first mammalian cell-only strain was included in Flucelvax^®^ Quadrivalent in 2017. A sequence analysis of the viruses selected for inclusion in this vaccine (*n* = 15 vaccine strains, containing both hemagglutinin and neuraminidase) demonstrated that no mutations occur in the antigenic sites of either hemagglutinin or neuraminidase, indicating that *cell* adaptation does not occur during production of this cell-based vaccine. The development of this now entirely mammalian-based vaccine system, which incorporates both hemagglutinin and neuraminidase, ensures that the significant protective antigens are equivalent to the strains recommended by the World Health Organization (WHO) in both amino acid sequence and glycosylation pattern. The inclusion of both proteins in a vaccine may provide an advantage over recombinant vaccines containing hemagglutinin alone. Findings from real world effectiveness studies support the use of cell-based influenza vaccines.

## 1. Introduction

Seasonal influenza vaccines are the primary means of reducing the global burden of influenza—a disease that annually causes approximately 300,000–650,000 deaths worldwide and substantial economic burden in both low and high income countries [1,2,3,4]. In the US, the estimated total economic burden due to influenza is $11.2 billion, including $3.2 billion in direct healthcare costs and $8.0 billion in lost productivity [2]. Studies of influenza vaccine cost effectiveness consistently show savings of $10,000–$50,000 per influenza-related outcome in middle and high income countries [5].

Influenza viruses are single-stranded, segmented RNA viruses with low RNA polymerase fidelity. As such, the influenza virus genome mutates at a much higher rate than genomes consisting of double-stranded DNA [6]. As influenza virus replicates in humans and animals, selection pressure frequently leads to changes in the influenza viral surface glycoproteins—more often in hemagglutinin (HA) than neuraminidase (NA); such mutations can enable subsequent generations of influenza viruses to escape human immune responses, alter receptor binding and affinity and add or remove glycosylation sites. The rapid, ongoing evolution of HA and NA is known as antigenic drift. HA and NA amino acid substitutions due to antigenic drift among circulating viruses can have variable impacts on the antigenic relatedness between circulating and vaccine viruses (e.g., HA head mutations tend to have a greater impact than stalk mutations). In addition, pandemic influenza viruses emerge due to antigenic shift, in which two separate virus strains exchange genetic material in a process called reassortment, creating a novel influenza virus such as the novel A(H1N1) virus that caused the 2009 influenza pandemic [7,8].

**Figure 1:**
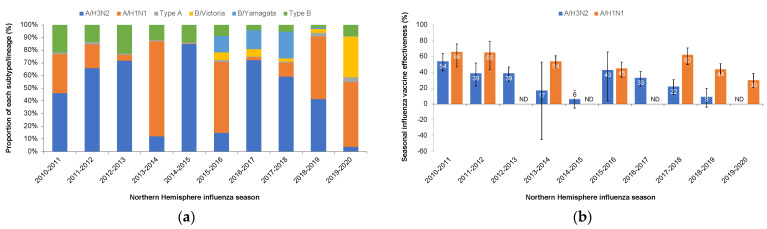

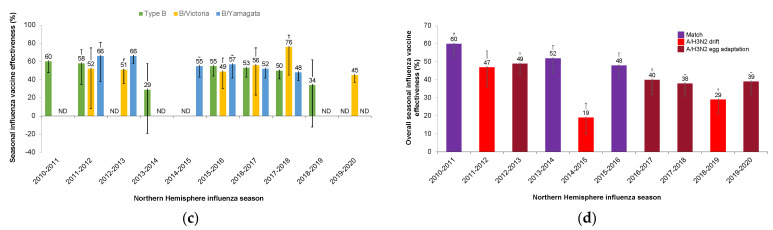
Seasonal influenza vaccine effectiveness in the US as estimated by the Centers for Disease Control and Prevention (CDC) [15,16,17,18,19,20,21,22,23,24,25]. (**a**) Proportions of identified virus types, subtypes, and lineages by year. (**b**) Adjusted vaccine effectiveness for influenza A strains. (**c**) Adjusted vaccine effectiveness for influenza B strains. (**d**) Adjusted overall seasonal effectiveness. Error bars for (**b**–**d**) indicate adjusted 95% confidence interval. ND, no data.

Rapid viral evolution and antigenic drift necessitates annual review and often reformulation of influenza vaccines. Mid-season and end-of season analyses of vaccine effectiveness inform vaccine recommendations by the WHO, particularly as a reduction in effectiveness may indicate antigenic drift. Notably, a number of other factors besides antigenic drift also contribute to the measurement of effectiveness of influenza vaccines: (i) the prevalence of a subtype or lineage; (ii) the immune response of an individual to the vaccine, which is influenced by vaccine dose, immunogenicity, inclusion of adjuvant, prior immunity, and (iii) match of the vaccine antigen to the recommended strain. Embryonated hens’ eggs have been the substrate for influenza vaccine production for over 60 years. Amino acid changes of the HA and NA sequences during serial passage in eggs is common and enables more efficient binding and thus growth in this platform (termed ‘egg adaptation’). Egg adaptation can affect antigenicity, which in turn affects vaccine effectiveness [9]. Between the 2010–2011 and 2019–2020 seasons, overall influenza vaccine effectiveness as estimated by the US Centers for Disease Control and Prevention (CDC) ranged between 19 and 60% in the US after adjustment for study site, age, sex, underlying medical conditions, and days from illness onset to enrolment [10]. Analysis of vaccine effectiveness of the influenza B component of the vaccine shows relative consistency; cross-reactivity of the antibody responses between the lineages may contribute [11]. Notably, in the 2014–2015, 2017–2018, and 2018–2019 Northern Hemisphere seasons, when A(H3N2) circulated at high levels, the effectiveness of A(H3N2) and the adjusted overall effectiveness dropped. In addition to antigenic drift, egg adaptation during vaccine manufacture contributed to vaccine and circulating virus mismatch during this 10-season period (Figure 1) [8,12,13]. Depending on the prevalence of the affected subtype or circulating lineage, the impact of egg-adaptation on vaccine effectiveness is unpredictable and varies season to season [14].

**Figure 2:**
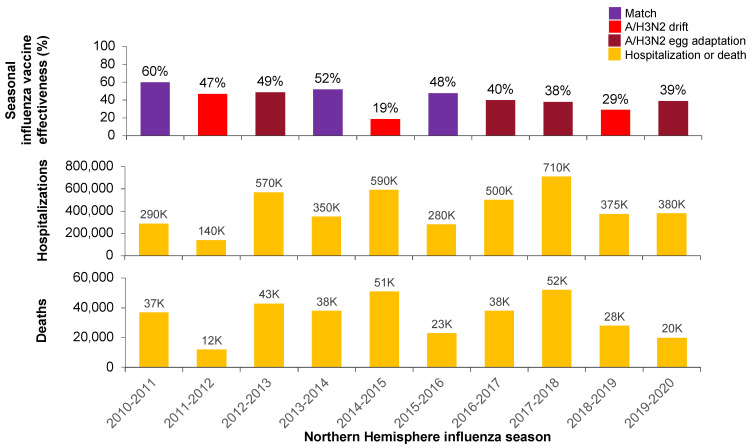
CDC-adjusted overall vaccine effectiveness estimates and documented A/H3N2 antigenic match or mismatch each season, as shown in Figure 1 (**top**), with numbers of US hospitalizations (**middle**) and deaths (**bottom**) due to influenza [15,16,17,18,19,20,21,22,23,24,26,27,28,29].

Mismatched seasons tend to be associated with higher rates of hospitalizations and deaths due to influenza, even in years with higher vaccine effectiveness (Figure 2) [15,16,17,18,19,20,21,22,23,24,26,27,28,29]. Egg adaptation has previously been documented to occur in up to 55% of H3N2 strains [30]. However, influenza burden of disease in any given season is driven by a complex mix of factors that also includes virulence of the predominant strain(s), influenza vaccine coverage, the immunogenicity of the vaccine, the level of vaccines response in various population groups (e.g., the elderly) and imprinting effects [31,32].

A recent expert consensus report suggested that egg adaptation reduces vaccine match by 7 to 21% and may reduce vaccine effectiveness by 4 to 16%, similar to antigenic drift of circulating influenza viruses, which reduces vaccine match and effectiveness by an estimated 8 to 24% and 5 to 20%, respectively [33]. Whilst prediction of virus evolution and antigenic drift is complex, vaccines which do not have the burden of egg adaptation are currently available. Mammalian cell-based vaccines have been licensed since 2012 and recombinant influenza vaccines, containing only the HA of influenza, have been licensed since 2013. We have previously published a detailed review of non-egg influenza vaccines [34,35]. Herein, we discuss the benefit of non-egg influenza vaccine strategies available at this time to improve vaccine effectiveness.

## 2. Mutations in Cell-Based and Egg-Based CVV Isolates and Implications for Influenza Vaccine Effectiveness

### 2.1. CVV Isolation and Sequencing Studies

The Global Influenza Surveillance and Response System (GISRS) assesses the antigenicity of currently circulating influenza viruses for match to the current influenza vaccine. To enable viruses for influenza vaccine manufacture, viruses from human original clinical samples (OCS) are isolated in embryonated hen’s eggs or in qualified manufacturing cells, such as MDCK 33016PF cells. These candidate vaccine viruses (CVVs) are assessed genetically and antigenically for suitability. Viruses isolated on both platforms may be reassorted with high growth manufacturing non-egg strains to generate reassortant viruses. Reassorted viruses contain the HA and NA proteins of the currently circulating strain and the internal proteins of the high growth parental strain to generate viruses suitable for large scale manufacture. Since the 2017–2018 season, mammalian cell-based CVVs have been used to generate the MDCK cell-based vaccine, with an entirely cell-based vaccine from 2019–2020 onwards. Recombinant influenza vaccines derive their sequences from cell-based CVVs; however this is limited to the HA only (NA is not included in these vaccines) [36].

A review of CVV isolation data illustrates important differences between mammalian cell-based and egg-based vaccine production methods. A retrospective study compared the isolation of influenza-positive human clinical respiratory samples in eggs and MDCK 33016PF cells as part of routine influenza surveillance at the Melbourne WHO Collaborating Centre for Reference and Research on Influenza between 2008 and 2020. The isolation rates of CVVs following growth in eggs and MDCK cells were evaluated to compare the susceptibility of each substrate in supporting efficient growth of human influenza viruses. Sequence analyses were also conducted to characterize resulting mutations that emerged [37]. The number of viral isolates was substantially greater when MDCK cells were used as the substrate for isolation when compared to eggs as the substrate. Furthermore, the mutation rate was higher in egg grown vs. cell grown CVVs. Out of 895 OCS inoculated into both the egg and MDCK cell substrates, twice as many viruses were isolated from cells as from eggs (81.0 vs. 40.6%; *p* < 0.00001) across virus strains. Moreover, year-to-year variability in isolation rates was greater in eggs for A/H1N1 and B/Yamagata strains. Between 2011 and 2017, different A/H3N2 clades were more readily isolated from MDCK cells than from eggs, although there were no differences in the rate of A/H3N2 clade isolation rates in the early and later years of the study. In summary, 4 out of 5 viruses inoculated into mammalian cells grew, compared with only 2 of 5 viruses inoculated into eggs. In addition, unique, nonsynonymous mutations in the HA gene were observed in 93% of viruses passaged in eggs compared with only 11% of viruses passaged in the cell line, representing an 8.5-fold difference in the incidence of nonsynonymous mutations between eggs and cells [37]. This suggests that, despite up to 5 passages in eggs, the replication efficiency of human-derived CVVs remained low in eggs across multiple years, enabling the selection of adaptive changes in egg-grown CVVs to enhance virus replication in the egg substrate. These observations reinforce the pitfalls of egg-based CVV selection and eventual vaccine production.

Although mutations in cells were rare (detected in 18/166 viruses passaged in cells), nonsynonymous amino acid changes were reported when CVVs were grown in MDCK cells. Mutations that did occur during growth in the MDCK cell substrate were distributed across the entire HA protein, with half of the amino acids sites with changes observed located in the globular head and other half located in the stalk/HA2 and only 35% of changes in the HA observed in the antigenic or receptor binding sites. Mutations were only common for two viruses. This is consistent with random mutations that may arise due to a lack of proofreading by the viral RNA polymerase. Hence, the observed mutations reflect the sequence variability observed with replication of circulating human viruses and are not specific to virus growth in MDCK cells. In contrast, 72% of amino acid sites changed in egg passaged viruses were located in the globular head of the HA, and 63% of mutations observed in the HA of viruses passaged in eggs mapped to the antigenic and receptor binding sites, which are subject to selective pressure from the avian growth substrate. These mutations were frequent and repeatedly occurred at specific sites in multiple viruses within these protein domains, enabling enhanced growth in eggs [37].

In a sequence analysis conducted at CSL Seqirus, investigators compared the surface domains of HA and NA isolated from patient-derived OCS, CVVs minimally passaged through MDCK cultures at WHO Collaborating Centres, and commercial manufacturing strains (ie, the working seed virus [WSV]) obtained after MDCK cell passage at Seqirus laboratories (Table 1 and Table S1) [38]. Sequence data was available for Northern and Southern Hemisphere seasons, beginning in Northern Hemisphere 2018–2019 through the Northern Hemisphere 2022–2023 season. Within these seasons, in the comparison between OCS and WHO-passaged viruses, sequence data were available for 13 of 16 manufacturing strains, and in the comparison between the OCS or WHO-passaged strains and the final working seed virus, sequence data were available for 14 of 16 manufacturing strains. Across the 4 influenza virus types, only two, non-antigenic site, sequence deviations were detected. In the first instance, a mixed base, S12S/E, in the transmembrane domain of NA of A/North Carolina/4/2016 (A/H3N2) was observed in the comparison between the OCS and the WHO-passaged viruses; the affected site is not antigenic. The second sequence deviation was observed in the comparison between the OCS and the WVS and was an HA protein sequence mixture at E224E/K in A/Delaware/55/2019 (A/H1N1). Position 224 is in the 220-loop, part of the receptor binding region. It is yet to be determined whether this affects antigenicity. The E224E/K mixture has also been reported in the same virus passaged in MDCK cells (C1, C2) and eggs (E1, E2, E3) by the CDC; submitted sequences to GISAID [39]), indicating that this position is unstable and most likely reflects the circulating strains; as such, it was included for manufacturing. In summary, no cell-specific adaptive mutations have been observed and viruses passaged in MDCK cells are representative of those isolated in humans.

### 2.2. Impact of Antigenic Mutations on Vaccine Effects

Evidence from real-world, observational studies and randomized controlled trials supports the negative impact of egg adaptation on the level of protection offered by influenza vaccines. Across multiple real-world observational studies spanning different seasons comparing the effectiveness of egg- and cell-based influenza vaccines, cell-based vaccines have generally been found to be more effective than egg-based vaccines, particularly in adults 18–64 years of age [40,41,42,43,44,45,46,47,48,49,50]. A recent meta-analysis of observational studies conducted during the 2017–2018 and 2018–2019 influenza seasons highlights the potential impact of egg adaptation vs. antigenic drift on vaccine effectiveness [40]. The 2017–2018 season was characterized by significant A/H3N2 egg adaptation and was also a high-severity influenza season [22]. During this season, cell-based influenza vaccines were found to be 11% (95% CI, 8–14%) more effective than egg-based vaccines in the prevention of influenza-related outcomes in adults ≥18 years of age, and the relative risk of influenza-related hospitalizations was reduced by 8% (adjusted relative risk, 0.92 [95% CI, 0.88–0.97; *p* = 0.002]) [40]. During the 2018–2019 season, A/H3N2 antigenic drift, but not egg adaptation, was observed, and the overall vaccine effectiveness was lower than in the previous season (Figure 1) [23]. No significant difference in effectiveness was observed in the overall adult population during the 2018–2019 season, but the relative effectiveness of cell- vs. egg-based influenza vaccines was 6% (95% CI, 5–8%) in adults 18–64 years of age [40]. Similarly, individual observational studies from the 2019–2020 season all reported a benefit of cell-based vaccines over egg-based vaccines for individuals younger than 65 years (estimates ranged from 5.3 [95% CI, 0.5–9.9%] to 12.2% [95% CI, 7.5–16.6%], but not for adults aged 65 and older [41,42,51,52,53]. This result is consistent with observations that the immune responses of older adults are impaired due to immunosenescence, and adjuvanted or high-dose influenza vaccines are recommended to enhance immune responses in this population [32].

The impact of egg-adaptation on influenza vaccines may also have long-lasting implications for immunity against influenza viruses over time, especially in the very young. A US CDC study that examined pre- and post-vaccination microneutralization geometric mean titers found that when children received an egg-adapted vaccine as their first exposure to influenza antigen, they mounted antibody responses largely targeting undesirable egg-adapted epitopes that were absent on circulating viruses. This may direct future antibody responses towards egg-adapted epitopes instead of wildtype virus epitopes after subsequent influenza vaccinations. In contrast, individuals who were likely first primed by natural infection with the A/H3N2 virus mounted antibody responses to epitopes that are shared with cell-propagated viruses, even when egg-based vaccines were subsequently received [32]. This study highlights the importance of alternatives to egg-based vaccines to protect the pediatric population long-term as they age.

The 2014–2015 influenza season was marked by low overall vaccine effectiveness and A/H3N2 antigenic drift [19]. In a randomized, controlled trial conducted during that season in adults ≥50 years of age, the recombinant influenza vaccine demonstrated 30% (95% CI, 10–47%) greater efficacy at preventing infections than the egg-based comparator (infection rates determined by reverse-transcriptase polymerase-chain-reaction (RT-PCR)–confirmation). In subgroup analyses, the results were significant in adults 50–64 years of age (relative vaccine efficacy 42% [95% CI, 15–61%]) but not in participants ≥65 years of age (17% [−20 to 43%]) [54]. Since recombinant HA alone is less antigenic to cell- or egg-derived HA antigens, the recombinant influenza vaccine contains 45 μg of HA per strain—3 times more than the standard dose, egg-based comparator used in this study. Thus, it is difficult to differentiate the respective contributions of the vaccine’s higher HA antigen content and/or lack of egg-adaptive changes to the higher relative efficacy [34,54,55].

## 3. Roles of NA and Glycosylation in Immunity and Potential for Future Vaccine Design

### 3.1. Complementary Roles of HA and NA in Influenza Infection and Disease

HA has historically been considered the primary target of the body’s immune response, and thus influenza vaccines have historically been engineered to elicit anti-HA antibodies. A type I transmembrane protein, HA is more abundant than NA and is directly involved in viral binding to host cell membranes. Host antibodies prevent viral attachment and entry into cells by blocking the HA receptor-binding site from interacting with sialic acids on the host cell surface. As described earlier, evolutionary pressure to evade host antibodies promotes the rapid changes in HA that necessitate annual vaccine reformulation and leads to frequent mismatches between vaccine and circulating influenza viruses [56,57].

In contrast, NA is a more stable, tetrameric type II transmembrane protein [56,58,59]. It is more conserved than HA, with a slower rate of mutations [56,57,58]. NA assists with host cell entry by cleaving terminal sialic acid from glycans on the host cell surface, which permits HA to bypass mucosal barriers and enter the host cell. After viral replication within the host, the actions of NA promote release and spread of newly made viral particles [56].

Despite the historic focus on HA, serum analysis has indicated that, despite only being present in small amounts on each virion, NA may be the most immunogenic of all influenza proteins [59]. Although NA antibodies do not directly prevent viral entry into host cells, they impair NA’s actions that facilitate HA binding to host cell receptors. NA antibodies also prevent virus budding and release from cells and might also aid in viral clearance [58]. NA antibodies dramatically reduce growth of viral plaques in vitro and clinical symptoms of influenza in animal models and human subjects [56]. NA also appears to provide cross-protection from heterologous influenza strains, and natural infection induces NA-reactive B cells, suggesting a role in long-term immunity to influenza [58]. Pre-existing NA antibodies have also been associated with shorter duration of viral shedding and a reduction in the duration of viral symptoms [60].

Overall, the actions of HA result in influenza infection, whereas NA plays a more important role in clinical disease, viral shedding, and spread of influenza to other individuals [56,57,58]. For these reasons, HA has been the focus of influenza prophylaxis with vaccines, whereas NA inhibition forms the basis for influenza treatment with antiviral therapies such as oseltamivir, zanamivir, and peramivir [61]. NA has potential as a vaccine target, but currently available vaccines do not contain standardized amounts of NA, and the process of inactivation may impair binding of NA-reactive antibodies in vaccinated subjects [58]. Nevertheless, despite not containing a standard amount of NA, current vaccines have shown that they are capable of stimulating an immune response to NA [62]. Challenges with NA inhibition assay methodology have also hindered targeting of NA with vaccines [57]. Active research is ongoing to overcome these challenges and develop effective vaccines with standardized NA content.

### 3.2. Impact of Glycosylation on Interactions between Viruses and Human Cells

**Figure 3:**
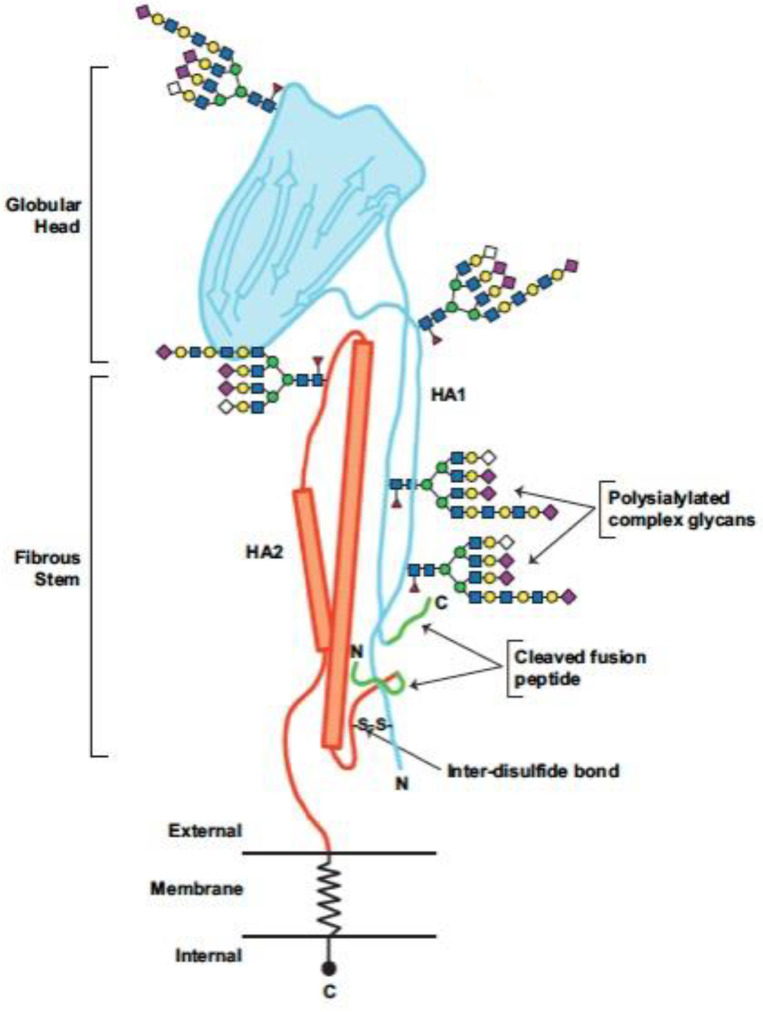
Influenza HA protein is a heterodimer comprising HA1 (turquoise) and HA2 (orange) linked though an inter-disulfide (S–S) bond and containing complex-type sialylated N-linked glycans. Cleaved fusion peptides (green) eliminate unique HA epitopes, and complex glycans mask unique epitopes on HA. Modified and reprinted with permission from Arunachalam et al. NPJ Vaccines 2021;6:144 [65]. Creative commons license: https://creativecommons.org/licenses/by/4.0/, accessed on 1 October 2022.

Glycosylation (or the addition of carbohydrates known as glycans to the functional groups of molecules) is necessary for protein folding across phyla. In viruses, mutations that create new *N*-glycosylation sites in the HA1 globular domain (Figure 3) commonly occur in response to host immune responses. HA2 is more conserved, with a lower mutation rate, than HA1. Since their first appearances in 1918 and 1968, respectively, A/H1N1 and A/H3N2 have acquired glycans in the globular head and stem regions of the HA molecule. Over time, the addition of more and more complex glycosylation sites shields antigenic sites, often neutralizing host antibodies. Depending on their location, the additional glycans may also make the HA more susceptible to lectin-based host defense systems [63,64,65].

The influence of glycosylation on vaccine effectiveness is not well understood. Inactivated vaccines passaged in eggs or mammalian cells retain the complex HA glycans found in circulating influenza strains and CVVs. In contrast, expression of antigen from insect cell lines results in a recombinant HA which contains smaller, simpler paucimannose glycans [65]. Some authors have proposed that the simpler glycan structures may improve antibody responses and cross-protection from heterologous strains; however, these studies have not compared immunogenicity with equivalent amounts of antigen during vaccination (egg-based vaccine [Fluzone^®^, Sanofi] contained 15 μg HA per strain, MDCK cell-based vaccine [Flucelvax] contained 15 μg HA per strain, and recombinant vaccine [Flublok] contained 45 μg HA per strain) [65,66]. Antibody responses (hemagglutination inhibition responses and affinity binding to HA1) to A/H1N1 and A/H3N2 HA were observed to be higher in subjects who received the recombinant influenza vaccine where three-fold more antigen was delivered compared to recipients of the comparator cell and egg vaccines [66]. It should be noted that the composition of the vaccine matrix will also affect immunogenicity, whereby soluble vaccines, unless the proteins are complexed in some manner, are generally less immunogenic than antigens that are presented to the immune system as protein arrays, such as whole viruses, or split virus particles [67]. However, the elevated antibody responses did not correlate with any significant differences between the vaccines in terms of binding to HA1 of B strains, HA2 binding, cross-neutralization of heterosubtypic strains, or antibody-affinity maturation, wherein a vaccine induces the generation of high-affinity antibodies and long-term plasma cells and memory B cells, a key aspect of long-term immunity. All 3 vaccines similarly elicited significant HA1-antibody affinity maturation, which declined similarly across vaccine platforms over the ensuing year [66]. These findings would suggest that the expression of simpler glycosylation patterns on HA associated with the use of insect cell lines within recombinant vaccines may not significantly impact their immunogenicity, particularly once the amount of antigen delivered has been taken into consideration.

Glycosylation of human immune system proteins also has an impact on vaccine response, and in future might be considered in the selection of vaccine recipients. Findings from a recent study suggest that postvaccination protection is partly dependent on modulation of glycosylation by the human immune system [68].

## 4. Conclusions

Egg-based influenza vaccines remain a staple of influenza prevention efforts, but egg adaptation continues to contribute to reduced vaccine effectiveness. Newer vaccine technologies avoid problems with not only egg adaptation but also the structural and logistical challenges inherent in the annual production of millions of high-quality embryonated eggs. Cell-based vaccines produced using mammalian (MDCK) or insect (baculovirus recombinant) cell lines offer the potential for improving the antigenic match between vaccine and circulating virus strains. These newer platforms may also increase the speed of response to pandemic influenza as well as changes in circulating seasonal strains, yet questions have arisen regarding cell and baculovirus/insect-based vaccine platforms with regard to how representative the antigen delivered is when compared to circulating strains. 

The risk of mutations in the antigenic site of HA for both mammalian and recombinant approaches is very low. In MDCK cell lines, no adaptative mutations have been documented and where occasional mutations are observed, these are random and stochastic in nature, are not driven by selective pressure during culture. Furthermore, the WHO and commercial manufacturers have careful screening protocols in place to ensure CVVs and seed viruses that are best matched to circulating strains are included in production. For cell based vaccines, the sequence of cell isolated viruses are actively reviewed to ensure that there are no random mutations that could affect the antigenicity of the vaccine.

With respect to antigen presentation, the use of mammalian culture systems ensures that the antigen retains a similar glycosylation profile as that of the circulating human seasonal viruses. A mammalian system is also the optimal approach for a pandemic situation as it contains the complex glycosylation that may be present on pre-pandemic viruses once human-to-human transmission is maintained. For baculovirus-expressed HA from insect cell lines, the presentation of antigen is clearly differentiated from that present on circulating viruses due to inherent differences in glycosylation patterns. Mammalian cells retain the complex HA glycans found in circulating influenza strains and CVVs whereas insect cells are limited to the use of smaller, simpler paucimannose glycans. The impact of these glycosylation differences on vaccine effectiveness remains unclear. Although some comparisons between recombinant and egg and cell based vaccines have been undertaken, these studies have typically utilized 3-fold more recombinant antigen than egg and cell based vaccines to deliver similar levels of vaccine immunogenicity.

The future of influenza vaccines may lie in efforts to incorporate NA into vaccines in a way that preserves the robust, long-term immune responses to NA observed in individuals naturally infected with influenza. In addition, a better understanding of the impact of glycosylation of viral HA as well as human immune system proteins is needed to further refine vaccine design.

## Figures and Tables

**Table 1:** Comparison of sequence data for HA and NA isolated from virus samples obtained at different stages in the manufacturing process [38] ^a^.

Influenza Virus Type	OCS vs. MDCK ^b^		OCS/MDCK vs. WVS ^b^	
	HA	NA	HA	NA
A/H1N1	5/5 identical	5/5 identical	4/5 identical ^c^	5/5 identical
A/H3N2	5/5 identical	4/5 identical ^d^	5/5 identical	5/5 identical
B/Victoria	2/2 identical	2/2 identical	3/3 identical	3/3 identical
B/Yamagata	1/1 identical	1/1 identical	1/1 identical	1/1 identical

^a^ During manufacturing, viral genes are sequenced from patient-derived OCS. Viruses are then grown in WHO-passaged MDCK cells, which in turn are used as the WSV for vaccine production (i.e., OCS → MDCK → WVS). ^b^ Number of identical sequences/number of influenza viruses. ^c^ Mixed base corresponding to an unstable amino acid identified on multiple platforms. ^d^ Mixed base observed in the transmembrane domain of NA (amino acids 7–29), which is not an antigenic site. HA = hemagglutinin; MDCK = Madin-Darby canine kidney cell line, passaged at WHO Collaborating Centres; NA = neuraminidase; OCS = original clinical sample (patient-derived); WSV = working seed virus, passaged at Seqirus laboratories for commercial vaccine production.

## Data Availability

Sequence analysis of the working seed viruses see GISAID influenza sequence repository.

## Supplementary Materials

The following supporting information can be downloaded at: https://www.mdpi.com/article/10.3390/vaccines11010052/s1, Table S1. Detailed sequencing data from Seqirus sequence analysis.
