# Correction: Rockman et al. Cell-Based Manufacturing Technology Increases Antigenic Match of Influenza Vaccine and Results in Improved Effectiveness. *Vaccines* 2023, *11*, 52

**DOI:** 10.3390/vaccines11121839

**Published:** 2023-12-11

**Authors:** Steven Rockman, Karen Laurie, Chi Ong, Sankarasubramanian Rajaram, Ian McGovern, Vy Tran, John Youhanna

**Affiliations:** 1 CSL Seqirus Ltd., Parkville, VIC 3050, Australia; 2 Department of Immunology and Microbiology, The University of Melbourne, Parkville, VIC 3050, Australia; 3 CSL Seqirus Ltd., Maidenhead SL6, UK; 4 CSL Seqirus Ltd., Summit, NJ 07901, USA; 5 CSL Seqirus Ltd., Kirkland, QC H9H 4M7, Canada

The authors would like to make the following corrections to this published paper [1]. An incorrect color was assigned to season effectiveness in Figure 1d and Figure 2, due to a line color error that occurred transferring the figure from a graphics program to a word processing format. The color of the last column in Figure 1d and the color of the last column in Figure 2 (top) were both corrected. The corrected figures are shown below:

The authors apologize for any inconvenience caused and state that the scientific conclusions are unaffected. The original article has been updated.

## Figures and Tables

**Figure 1:**
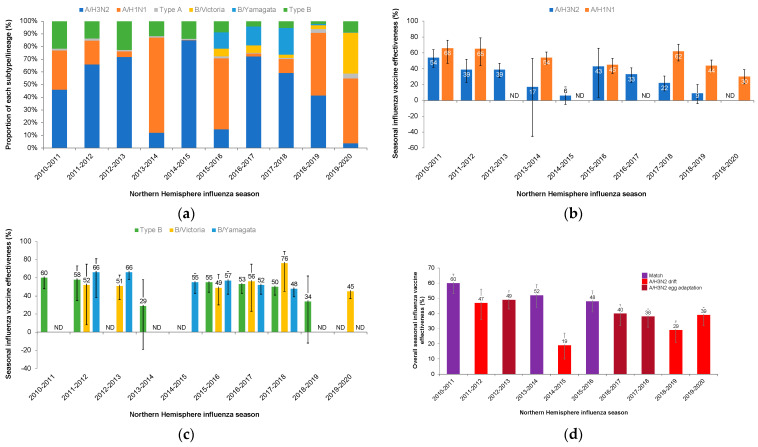
Seasonal influenza vaccine effectiveness in the US as estimated by the Centers for Disease Control and Prevention (CDC) [15–25]. (**a**) Proportions of identified virus types, subtypes, and lineages by year. (**b**) Adjusted vaccine effectiveness for influenza A strains. (**c**) Adjusted vaccine effectiveness for influenza B strains. (**d**) Adjusted overall seasonal effectiveness. Error bars for (**b**–**d**) indicate adjusted 95% confidence interval. ND, no data.

**Figure 2:**
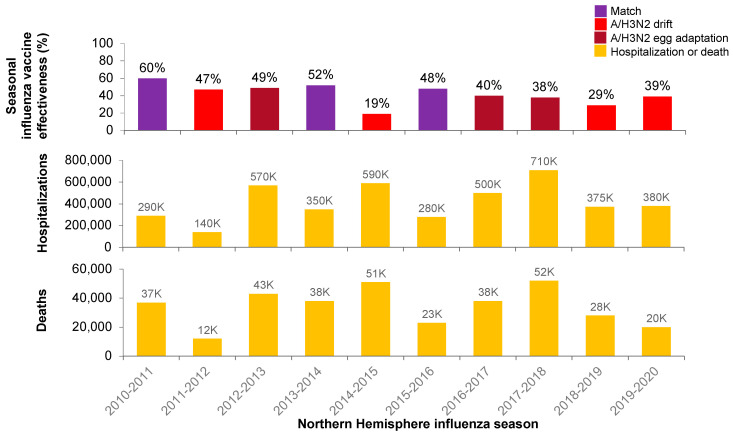
CDC-adjusted overall vaccine effectiveness estimates and documented A/H3N2 antigenic match or mismatch each season, as shown in Figure 1 (**top**), with numbers of US hospitalizations (**middle**) and deaths (**bottom**) due to influenza [15–24,26–29].
