# Supplementary material for: Cell-Based Manufacturing Technology Increases Antigenic Match of Influenza Vaccine and Results in Improved Effectiveness
Source: Vaccines (Basel). 2022 Dec 26;11(1):52. doi: 10.3390/vaccines11010052 (PMC9861528; doi:10.3390/vaccines11010052)
Supplement: Supplementary file 1 [file vaccines-11-00052-s001.zip › vaccines-2106959-supplementary.pdf]

Table S1. Detailed sequencing data from Seqirus sequence analysis.[a]

vaccine	WHO recommendation[b]	Seqirus vaccine strain[b]	OCS vs MDCK		WVS vs QCS/MDCK			HA aa	NA aa
			HA	NA	HA	NA	comparator		
**A(H1N1)**									
NH 19-20	A/Brisbane/02/2018	A/Idaho/07/2018	Identical	Identical	Identical	Identical	OCS	547	468
SH 2020	A/Brisbane/02/2018	A/Idaho/07/2018	Identical	Identical	Identical	Identical	OCS	547	468
NH 20-21	A/Hawaii/70/2019	A/Nebraska/14/2019	Identical	Identical	Identical	Identical	OCS	547	468
SH 2021	A/Wisconsin/588/2019	A/Delaware/55/2019	Identical	Identical	E224K/E[c]	Identical	OCS	547	468
NH 21-22	A/Wisconsin/588/2019	A/Washington/19/2020	Identical	Identical	Identical	Identical	OCS	547	468
SH 2022	A/Wisconsin/588/2019	A/Washington/19/2020	Identical	Identical	Identical	Identical	OCS	547	468
NH 22-23	A/Wisconsin/588/2019	A/Delaware/55/2019 CVR-45	Identical	Identical	Identical	Identical	OCS	547	468
**A(H3N2)**									
NH 17-18	A/Hong Kong/4801/2014	A/Singapore/GP2050/2015	no OCS	no OCS	no OCS, no WVS	no OCS, no WVS	N/A	549	469
SH 2018	A/Singapore/INFIMH-16-0019/2016	A/Singapore/GP2050/2015	no OCS	no OCS	no OCS, no WVS	no OCS, no WVS	N/A	549	469
NH 18-19	A/Singapore/INFIMH-16-0019/2016	A/North Carolina/04/2016	identical	S12S/G	no WVS	no WVS	N/A	549	469
SH 2019	A/Singapore/INFIMH-16-0019/2016	A/North Carolina/04/2016	identical	S12S/G	no WVS	no WVS	N/A	549	469
NH 19-20	A/Kansas/14/2017	A/Indiana/08/2018	Identical	Identical	Identical	Identical	OCS	549	469
SH 2020	A/Iowa/60/2018	A/Newcastle/82/2018	Identical	Identical	Identical	Identical	OCS	549	469
NH 20-21	A/Hong Kong/45/2019	A/Delaware/39/2019	Identical	Identical	Identical	Identical	OCS	549	469
SH 2021	A/Hong Kong/45/2019	A/Delaware/39/2019	Identical	Identical	Identical	Identical	OCS	549	469
NH 21-22	A/Cambodia/e0826360/2020	A/Tasmania/503/2020	no OCS	no OCS	Identical	Identical	QMC2	549	469
SH 2022	A/Darwin/6/2021	A/Darwin/11/2021	Identical	Identical	Identical	Identical	OCS	549	469
NH 22-23	A/Darwin/6/2021	A/Darwin/11/2021	Identical	Identical	Identical	Identical	OCS	549	469

**B/Victoria**									
NH 18-19	B/Colorado/06/2017	B/Iowa/06/2017	Identical	Identical	Identical	Identical	OCS	567	467
SH 2019	B/Colorado/06/2017	B/Iowa/06/2017	Identical	Identical	Identical	Identical	OCS	567	467
NH 19-20	B/Colorado/06/2017	B/Iowa/06/2017	Identical	Identical	Identical	Identical	OCS	567	467
SH 2020	B/Washington/02/2019	B/Darwin/7/2019	no OCS	no OCS	Identical	Identical	QMC2	567	467
NH 20-21	B/Washington/02/2019	B/Darwin/7/2019	no OCS	no OCS	Identical	Identical	QMC2	567	467
SH 2021	B/Washington/02/2019	B/Darwin/7/2019	no OCS	no OCS	Identical	Identical	QMC2	567	467
NH 21-22	B/Washington/02/2019	B/Darwin/7/2019	no OCS	no OCS	Identical	Identical	QMC2	567	467
SH 2022	B/Austria/1359417/2021	B/Singapore/WUH4618/2021	Identical	Identical	Identical	Identical	OCS	567	467
NH 22-23	B/Austria/1359417/2021	B/Singapore/WUH4618/2021	Identical	Identical	Identical	Identical	OCS	567	467
**B/Yamagata**									
NH 18-19	B/Phuket/3073/2013	B/Singapore/INFTT-16-0610/2016	Identical	Identical	Identical	Identical	OCS	574	466
SH 2019	B/Phuket/3073/2013	B/Singapore/INFTT-16-0610/2016	Identical	Identical	Identical	Identical	OCS	574	466
NH 19-20	B/Phuket/3073/2013	B/Singapore/INFTT-16-0610/2016	Identical	Identical	Identical	Identical	OCS	574	466
SH 2020	B/Phuket/3073/2013	B/Singapore/INFTT-16-0610/2016	Identical	Identical	Identical	Identical	OCS	574	466
NH 20-21	B/Phuket/3073/2013	B/Singapore/INFTT-16-0610/2016	Identical	Identical	Identical	Identical	OCS	574	466
SH 2021	B/Phuket/3073/2013	B/Singapore/INFTT-16-0610/2016	Identical	Identical	Identical	Identical	OCS	574	466
NH 21-22	B/Phuket/3073/2013	B/Singapore/INFTT-16-0610/2016	Identical	Identical	Identical	Identical	OCS	574	466
SH 2022	B/Phuket/3073/2013	B/Singapore/INFTT-16-0610/2016	Identical	Identical	Identical	Identical	OCS	574	466
NH 22-23	B/Phuket/3073/2013	B/Singapore/INFTT-16-0610/2016	Identical	Identical	Identical	Identical	OCS	574	466

[a] Sequencing data not available where specified (no OCS, no WVS).
[b] See nextstrain.org for detailed positioning of clades/subclades.
[c] H1 numbering without the signal peptide.
